# Dual-transcriptomic datasets evaluating the effect of the necrotrophic fungus *Alternaria brassicicola* on *Arabidopsis* germinating seeds

**DOI:** 10.1016/j.dib.2022.108530

**Published:** 2022-08-08

**Authors:** Mailen Ortega-Cuadros, Laurine Chir, Sophie Aligon, Tatiana Arias, Jerome Verdier, Philippe Grappin

**Affiliations:** a Institute of Biology, University of Antioquia, Calle 67 N° 53-108, Medellín 050010, Colombia; b Institut Agro, University Angers, INRAE, IRHS, SFR 4207 QuaSaV, Angers F-49000, France; c Marie Selby Botanical Gardens, Downtown Sarasota Campus, 1534 Mound Street, Sarasota, FL 34236, USA

**Keywords:** Germination, *Alternaria brassicicola*, *Arabidopsis thaliana*, Dual-transcriptomics, Plant-pathogen interaction, RNA-seq, Seed defense response

## Abstract

Many fungal pathogens are carried and transmitted by seeds. These pathogens affect germination and seed quality. Their transmission from the germinating seed to seedling causes many diseases in crops. Seed defense mechanisms during germination are poorly documented. RNA-seq experiments were used to describe the molecular mechanisms involved in seed interaction with a necrotrophic fungus. Here the *Arabidopsis thaliana/Alternaria brassicicola* pathosystem was used to perform dual-transcriptomic approach. *Arabidopsis thaliana* seeds and necrotrophic fungus transcripts were identified at critical germination and seedling establishment stages. Total RNA was extracted from healthy and infected germinating seeds and seedlings at 3, 6 and 10 days after sowing. Transcript libraries were made and sequenced, then fungal and plant short reads were mapped and quantified respectively against *Arabidopsis thaliana and Alternaria brassicicola* reference transcriptomes. This dual-transcriptomic approach revealed that 3409, 7506 and 8589 *Arabidopsis thaliana* genes showed a differential expression at respectevely 3, 6 and 10 days after sowing between healthy and infected seeds, including 1192 genes differentially expressed at the three studied stages. Moreover, in this experiement, we also identified the dynamic of the transcript changes occurring at the same stages in the necrotrophic fungus concomitantly during germination and seedling establishment.

## Specifications Table

**Table:** 

Subject	Agricultural and Biological Sciences
Specific subject area	Omics: Transcriptomics Plant Science: Plant Microbe Interaction
Type of data	Tables Figures
How the data were acquired	Seed germination were analyzed using the ScreenSeed automate [1]. Approximatively 15 mg of plant tissues were used for RNA isolation. An *Alternaria brassicicola* inoculum at 10^4^ conidia/mL was used for all infected conditions. Library construction and RNA paired-end sequencing (PE100, 40 M) was performed at Beijing Genomics Institute (BGI, https://www.bgi.com), Hong Kong using the DNA nanoball sequencing DNBseq™ technology. Raw data were analyzed using Salmon (version 0.14.1) [2], FastQC [3] and MultiQC tool [4] for mapping and quality control, DESeq2 [5] for differentially expression analysis and http://bioinformatics.psb.ugent.be/webtools/Venn/ for comparison of differential expressed genes (DEGs) in all conditions.
Data format	Filtered raw reads (FASTQ) Analyzed RNA-seq data files (counts and DEGs lists) Percentages of seed germination and infected seeds
Description of data collection	Healthy *Arabidopsis thaliana* seeds and *A. brassicicola* infected seeds were collected at three germination and post-germination time points (3, 6 and 10 days after sowing) from controlled growth chamber under a 16 h photoperiod at 22 °C/20 °C (day/night) and 70% relative humidity. RNA extracts were stored at −25 °C until sequencing. Sequence quality control was performed using FastQC [3] and MultiQC [4]. Filtered raw reads were mapped and quantified using the quasi-mapping alignment available in Salmon algorithm [2]. Fungal and plant reads were accordingly mapped to either *Arabidopsis* Araport 11 [6] or *A. brassicicola* Abra43 [7] reference transcriptomes.
Data source location	Institution: Growth chambers located at Institut de Recherche en Horticulture et Semences City: Beaucouzé Country: France GPS coordinates: 47°28′37.7″N 0°36′42.1″W
Data accessibility	Public Repository: Repository name: NCBI GEO Data identification number: GSE199977 Direct URL to data: https://www.ncbi.nlm.nih.gov/geo/query/acc.cgi?acc=GSE199977

## Value of the Data

• These data contribute to the understanding of interaction between a host plant and a necrotrophic fungus at the early stage of the plant's life cycles. This early developmental stage controlling transgenerational transmission of the fungal pathogen from seeds to the seedlings is not documented up to date.
• The data benefit both plant physiologists and pathologists.
  The dual-transcriptomic approach allows to describe transcriptional changes occuring concomitantly in *Arabidopsis* and *A. brassicicola*. This dataset allows the identification of candidate genes and molecular markers that reflect in one side seed defense response in *Arabidopsis* germinating seed and in other side virulence strategy of the necrotrofic fungus.
• This data set could be used for comparison of host/pathogen interactions at different developmental stages. Developmental kinetics at 3, 6, and 10 days after sowing, allows to describe interaction mechanisms which are specific to the germinating seed compared to those of the young seedling at the autotrophic. The response of the plant specifically induced by the infections can be characterized by a differential analysis of levels of expression between the infected and the uninfected samples.

## 1 Data Description

**Fig. 1:**
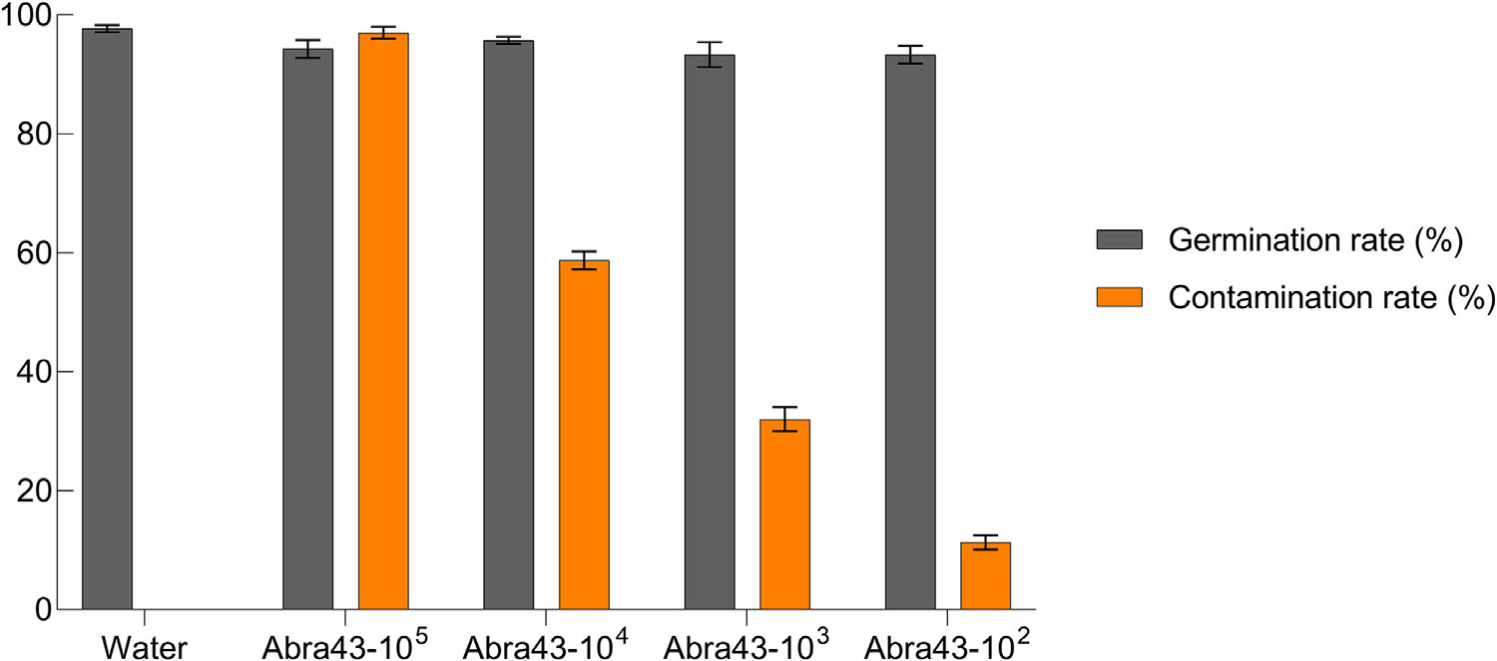
Seed germination of *Arabidopsis* and infection rates of *A. brassicicola* using four fungal inocula **(**and water as control). Figure made using GraphPad Prism 9, v. 9.3.1 (https://www.graphpad.com/).

**Fig. 2:**
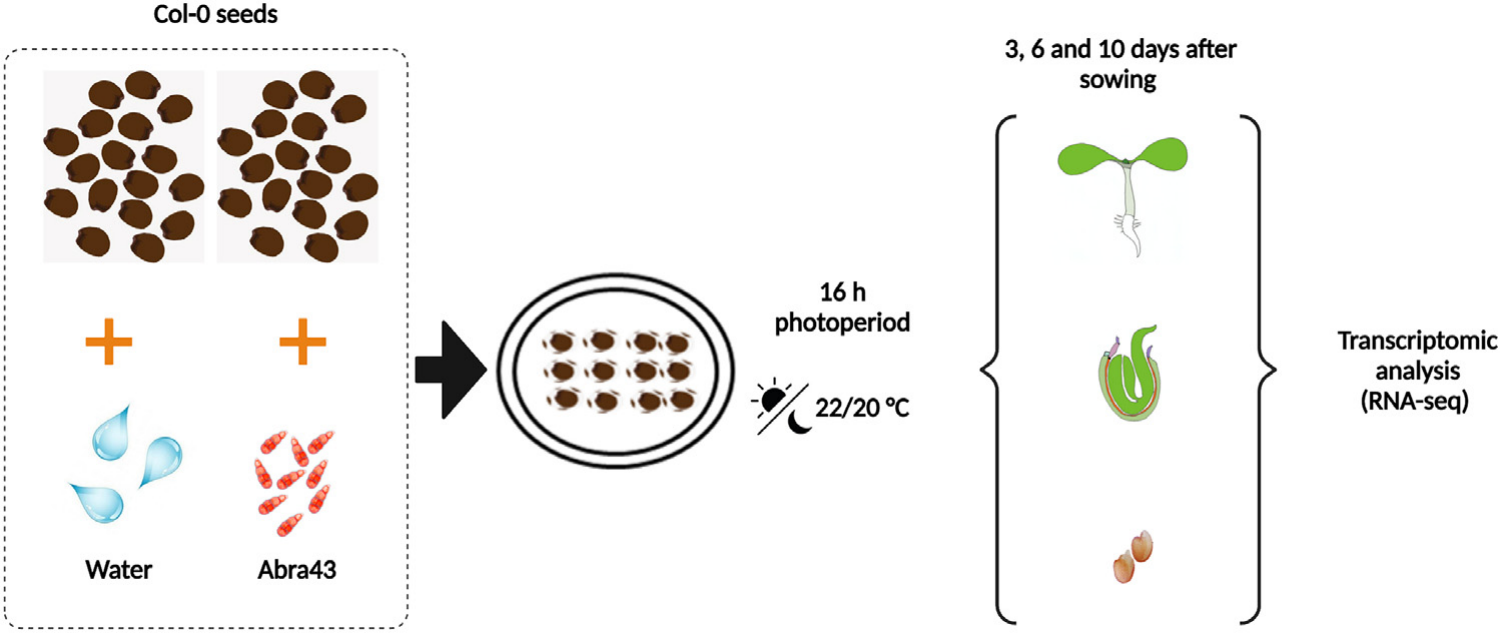
Experimental design used to obtain infected and healthy seed samples at 3, 6 and 10 days after sowing for RNA-seq analysis. These physiologial conditions have been chosen to identify the *A. brassicicola* and *Arabidopsis* (ecotype Col-0) seeds molecular interactions at the transcriptome level during seed-pathogen interaction*.* BioRender (https://biorender.com).

Plant pathogen interaction at germination and early post-germination stages need to be documented at the transcriptome level. Here is presented RNA sequencing for gene expression profiling upon *A. brassicicola* infection in germinating seed and at early seedling establishment using the pathosystem *Arabidopsis thaliana* (*Arabidopsis*)*/Alternaria brassicicola* (*A. brassicicola*). An optimal infection condition was determined with germination assay where seed germination and seed infection rates were scored for 10^2^, 10^3^, 10^4^, 10^5^ conidia/mL inoculum concentrations, respectively (Fig. 1). The optimal inoculum concentration of 10^4^ conidia/mL that did not affected seed germination and produced a significant seed infection rate was selected for the experimental conditions (Fig. 2) used in the RNA-seq analysis. All obtained sequence raw reads in *Arabidopsis* and in *A. Brassicicola* were deposited in the NCBI Sequence Read Archive (SRA) database under the repository name NCBI GEO with the data identification number GSRA99977 (https://www.ncbi.nlm.nih.gov/geo/query/acc.cgi?acc=GSE199977). Data were extracted from MultiQC [2] analysis (Fig. 2). The total number of filtered reads obtained after sequencing and the corresponding mapping rates using *Arabidopsis* publicly available transcriptomes (Araport 11) [6] and *A. brassicicola* [7] reference transcriptomes were obtained using Salmon algorithm [3]. Count files from *A. brassicicola* and *Arabidopsis* were for all three replicates and were used to identify differentially expressed genes between healthy and infected seeds at 3, 6 and 10 days after sowing. The pair-wise comparisons between healthy and infected host plant transcripts according to DEseq2 statistical analysis [5] identified 3409, 7506 and 8589 differentially expressed genes (DEGs) at 3, 6 and 10 days after sowing, respectively (Table S1).

**Fig. 3:**
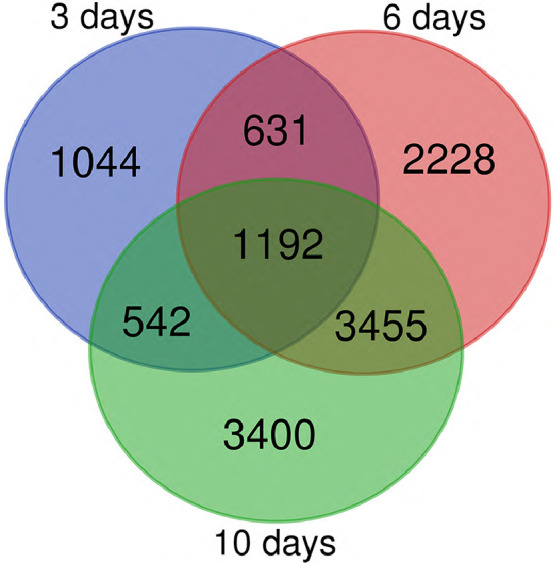
Venn diagram showing *Arabidopsis* uniquely differentially expressed genes (DEGs) (*i.e.* log_2_FC > 1 or < −1 and Benjamini-Hochberg score < 0.05) between healthy and *A. brassicicola* infected conditions at 3, 6 and 10 days after sowing. Also showing shared DEGs among conditions at the pre-germinative stage (3 days) compared to the stages of seedling establishment (6 days) and autotrophy (10 days) of the seedling.

A Venn diagram comparison of the three developmental stages (Fig. 3) exhibited 1192 common DEGs.

## 2 Experimental Design, Materials and Methods

### 2.1 Plant Material

*Arabidopsis* (Col-0 ecotype) mature seed lots were obtained from plants grown in a controlled climatic room at 19/20 °C, 16 h photoperiod of artificial light (150 µmol photons m^2^ s^−1^) and 70% relative humidity. Seeds (12 mg) were surface sterilised using 1 mL of 30% bleach treatment during 7 min, then followed by 7 min in 1 mL of 80% ethanol and five rinses in 1 mL of sterile deionized water. The seeds were dried for 5 h on a blotting paper in a Microbiological Safety Cabinet (SafeFAST Premium, FASTER, Cornaredo, MI, Italy).

### 2.2 Infection Assays

To select specific seed responses involved in the biotic interaction and not related to a germination defect, the seed inoculum concentrations were optimized to reach a maximal seed germination rate (Gmax). The Gmax as well as the infection rate of seeds of *Arabidopsis* ecotype Col-0 were evaluated to different concentrations of Abra43 *A. brassicicola* strain inoculum, *i.e.* 0, 10^2^, 10^3^, 10^4^, 10^5^ conidia/mL, respectively.

### 2.3 Germination Assays

For seed inoculation, 1 mL of the solution at the appropriate conidia concentration was added for one hour to 15 mg of seeds. The inoculated seeds were dried for 5 h on a blotting paper in a Microbiological Safety Cabinet (SafeFAST Premium, FAST-ER). Seed germination analyses were performed in microplates using the ScreenSeed automate according to the conditions described by Merieux et al. [1]. Incubation was performed inside a thermo-regulated incubator (Memmert ICP 750) regulated at 22 °C (±1 °C). Four replicates were measured in each condition analyzed and a minimum of 100 seeds per repeat was analyzed.

### 2.4 Sample Preparation

All sterilized seeds were inoculated with 10^4^ conidia/mL of *A. brassicicola*. The non-inoculated seeds were used as a control. Seeds infected or treated with water (non infected control seeds) were sowed in petri dishes containing 0.8% agarose (SIGMA) and cultures were incubated in a controlled growth chamber for 3, 6 and 10 days under a 16 h photoperiod (170 µmol photons m^2^ s^−1^) at 22 °C (light period)/20 °C (dark period) and a constant 70% relative humidity. 20 mg of seeds were used for each sample with three biological replicates per condition.

### 2.5 RNA Extraction and Sequencing

Seeds were collected at 3, 6 and 10 days after sowing. RNA extraction was performed using NucleoSpin® RNA Plus kit (Macherey-Nagel, Düren, Germany) according to the manufacturer's instructions. RNA quantification and quality were measured with a NanoDrop ND-100 (NanoDrop Technologies, DE, USA) and a 2100 Bioanalyzer (Agilent Technologies, Santa Clara, CA, USA) respectively. RNA samples were sent to Beijing Genomics Institute (BGI, https://www.bgi.com), Hong Kong for cDNA library construction paired-end sequencing (PE100, 40M) and sequencing using a DNA nanoball sequencing (DNBSEQ™) technology. DNBSEQ™ technology performed by BGI sequencing platform includes the single strand circular library construction, DNB generation and loading method, cPAS (combinatorial Probe Anchor Synthesis) sequencing technology.

### 2.6 RNA-seq Analyses

Mapping and quality control for raw reads was performed using a quasi-mapping alignment from Salmon, version 0.14.1 [2] and FastQC [3]. MultiQC tool [4] was used to summary all individual High-quality reads (Phred scores ≥ 35) from FastQC files. Filtered reads from seeds (infected or not) were mapped against the *Arabidopsis* Araport 11 [6] and the *A. brassicicola* Abra43 [7] reference genomes (Table 1). Differentially expressed genes (DEGs) between healthy and infected seeds were determined using DESeq2 [5]. Genes with log_2_FC > 1 or < − 1 and Benjamini-Hochberg score < 0.05 were considered as differentially expressed (Table S1). A Venn Diagram: http://bioinformatics.psb.ugent.be/webtools/Venn/ was performed for DEGs that were differentially expressed in all conditions was used (Fig. 3).

**Table 1:** Summary of mapping rate information obtained after mapping short reads using Salmon algorithm [2]. Col0: *Arabidopsis* seed; inoc: seed inoculated with *A. brassicicola*; water: seed without fungal inoculum; 3d, 6d, 10d: developmental stages of 3, 6 and 10 days after sowing respectively; REP: biological replicate.% Aligned:% Mapped reads; M Aligned: Mapped reads (millions); M Seqs: Total Sequences (millions).

		Mapping rate on *Arabidopsis* transcriptome		Mapping rate on *A. brassicicola* transcriptome	
Sample Name	M Seqs	% Aligned	M Aligned	% Aligned	M Aligned
Col0_inoc_3d_REP1	51.9	35.10%	18.2	44.40%	23.1
Col0_inoc_3d_REP2	48.5	23.10%	11.2	30.40%	14.8
Col0_inoc_3d_REP3	51.1	40.70%	20.8	41.00%	21
Col0_inoc_6d_REP1	50.9	20.30%	10.3	54.80%	27.9
Col0_inoc_6d_REP2	51	17.00%	8.7	57.00%	29
Col0_inoc_6d_REP3	51	19.10%	9.7	55.10%	28.1
Col0_inoc_10d_REP1	51.1	41.30%	21.1	39.30%	20.1
Col0_inoc_10d_REP2	51.2	57.30%	29.3	27.90%	14.3
Col0_inoc_10d_REP3	51.1	48.40%	24.7	34.70%	17.7
Col0_water_3d_REP1	25.9	97.10%	25.1	0.00%	0
Col0_water_3d_REP2	25.8	96.30%	24.9	0.00%	0
Col0_water_3d_REP3	26	96.50%	25	0.00%	0
Col0_water_6d_REP1	25.7	91.20%	23.4	0.00%	0
Col0_water_6d_REP2	25.6	94.20%	24.1	0.00%	0
Col0_water_6d_REP3	25.6	94.70%	24.2	0.00%	0
Col0_water_10d_REP1	25.8	96.00%	24.8	0.00%	0
Col0_water_10d_REP2	25.8	96.40%	24.9	0.00%	0
Col0_water_10d_REP3	25.8	96.20%	24.8	0.00%	0

## Ethics Statements

This work does not contain any studies with human or animal subjects*.*

## CRediT authorship contribution statement

**Mailen Ortega-Cuadros:** Conceptualization, Writing – review & editing, Supervision. **Laurine Chir:** Conceptualization, Visualization, Data curation. **Sophie Aligon:** Conceptualization, Visualization, Data curation. **Tatiana Arias:** Conceptualization, Writing – review & editing, Supervision. **Jerome Verdier:** Data curation, Formal analysis, Writing – review & editing, Supervision. **Philippe Grappin:** Funding acquisition, Visualization, Writing – review & editing, Supervision.

## Declaration of Competing Interest

The authors declare that they have no known competing financial interests or personal relationships that could have appeared to influence the work reported in this paper.

## Data Availability

Dual-transcritome analysis of germinating Arabidopsis seeds in response to necrotrophic fungus Alternaria brassicicola (Original data) (NCBI GEO).

## Appendix Supplementary materials

Image, application 1

Image, application 2

## References

[1] Merieux N., Cordier P., Wagner M.H., Ducournau S., Aligon S., Job D., Grappin P., Grappin E. (2021). ScreenSeed as a novel high throughput seed germination phenotyping method. Sci. Rep..

[2] Patro R., Duggal G., Love M.I., Irizarry R.A., Kingsford C. (2017). Salmon provides fast and bias-aware quantification of transcript expression. Nat. Methods.

[3] S. Andrews, F. Krueger, A. Segonds-Pichon, L. Biggins, C. Krueger, S. Wingett, FastQC. A quality control tool for high throughput sequence data, MA. Burlington: ScienceOpen, 370 (2010). https://www.bioinformatics.babraham.ac.uk/projects/fastqc/ (accessed March 22, 2022).

[4] Ewels P., Magnusson M., Lundin S., Käller M. (2016). MultiQC: summarize analysis results for multiple tools and samples in a single report. Bioinformatics.

[5] Love M.I., Huber W., Anders S. (2014). Moderated estimation of fold change and dispersion for RNA-seq data with DESeq2. Genome Biol..

[6] C.Y. Cheng, V. Krishnakumar, A.P. Chan, F. Thibaud‐Nissen, S. Schobel, C.D. Town, Araport11: a complete reannotation of the *Arabidopsis thaliana* reference genome, Plant J. 89 (2017) 789-804, doi:10.1111/tpj.13415. https://www.arabidopsis.org. Accessed March 26, 2022. 27862469

[7] Belmas E., Briand M., Kwasiborski A., Colou J., N'Guyen G., Iacomi B., Grappin P., Campion C., Simoneau P., Barret M., Guillemette T. (2018). Genome sequence of the necrotrophic plant pathogen *Alternaria brassicicola* Abra43. Genome Announc..

